# Investigation of a Standardized Qualitative Behaviour Assessment and Exploration of Potential Influencing Factors on the Emotional State of Dairy Calves

**DOI:** 10.3390/ani9100757

**Published:** 2019-10-02

**Authors:** Marta Brscic, Nina Dam Otten, Barbara Contiero, Marlene Katharina Kirchner

**Affiliations:** 1 Department of Animal Medicine, Production and Health, University of Padova, 35020 Legnaro (PD), Italy; marta.brscic@unipd.it (M.B.); barbara.contiero@unipd.it (B.C.); 2 Institute of Animal Welfare and Disease Control, Dep. Veterinary and Animal Sciences, University of Copenhagen, 1870 Frederiksberg C, Denmark; nio@sund.ku.dk

**Keywords:** calves, emotional state, organic, farm size, term list

## Abstract

**Simple Summary:**

Although welfare states of dairy calves are of public and scientific concern, no standardized protocol exists to assess the emotionality of these animals. Therefore, this study aimed at investigating and establishing a calf-specific term list for Qualitative Behavior Assessment (QBA), a technique that is already validated for assessing emotional states in many animal species. The statistically supported results showed that agreement can be reached among observers, terms showed varied results across farms, and evaluated emotional states could be linked to some explaining farm factors. Overall, results showed that calves have a neutral emotional state and profit from certain farm factors. However, we conclude that the assessment should be more widely used to gain more insight into calves’ welfare states and how their emotional state can be improved to a positive one.

**Abstract:**

Assessing emotional states of dairy calves is an essential part of welfare assessment, but standardized protocols are absent. The present study aims at assessing the emotional states of dairy calves and establishing a reliable standard procedure with Qualitative Behavioral Assessment (QBA) and 20 defined terms. Video material was used to compare multiple observer results. Further, live observations were performed on 49 dairy herds in Denmark and Italy. Principal Component Analysis (PCA) identified observer agreement and QBA dimensions (PC). For achieving overall welfare judgment, PC1-scores were turned into the Welfare Quality (WQ) criterion ‘Positive Emotional State’. Finally, farm factors’ influence on the WQ criterion was evaluated by mixed linear models. PCA summarized QBA descriptors as PC1 ‘Valence’ and PC2 ‘Arousal’ (explained variation 40.3% and 13.3%). The highest positive descriptor loadings on PC1 was Happy (0.92) and Nervous (0.72) on PC2. The WQ-criterion score (WQ-C12) was on average 51.1 ± 9.0 points (0: worst to 100: excellent state) and ‘Number of calves’, ‘Farming style’, and ‘Breed’ explained 18% of the variability of it. We conclude that the 20 terms achieved a high portion of explained variation providing a differentiated view on the emotional state of calves. The defined term list proved to need good training for observer agreement.

## 1. Introduction

Positive indicators in animal welfare assessment schemes are still limited although, nowadays, essential in many ways. Qualitative Behavioral Assessment (QBA) focuses on animals’ demeanor and body language to identify the underlying emotional state. QBA is one possibility which is frequently used to assess animals’ emotional states on-farm and in experiments, using either a free choice profiling (FCP) [1] or term lists as behavioral descriptors [2]. Validity aspects of QBA were investigated in various animal species and situations such as the ability to picture anxiety in pigs [3], in relation to farm factors in veal calves [4], or stress levels during transport in sheep [5]. Additionally, reliability was proven in some studies and species, such as intra-day variation of QBA in dairy cows [6]. After investigations on observer reliability in dairy cows, fattening cattle, and veal calves [2], QBA was implemented as a measure for the criteria ‘positive emotional state’ (WQ-C12) in the Welfare Quality (WQ) assessment protocols for cattle, pigs, and chicken. QBA was used to address the criteria ‘positive emotional state’ (WQ-C12) [2] with specified terms for the respective livestock species, category, or types. In parallel to the development of these standardized lists in WQ, mathematical procedures were set in place to summarize a Principal Component Score (PC1). The potential range of this PC1 underwent an expert-based evaluation leading subsequently to a welfare interpretation of the QBA result into the WQ-C12, achieving points on a welfare state scale from 0 (worst) to 100 (excellent). Whenever establishing a QBA for new species, studies on reliability between observers and association with other factors, such as housing conditions or health state, were part of the preliminary studies. These studies aimed at validating the assessment method by looking at variation in the sample and at the potential of QBA to discriminate between each husbandry system. Several studies assessing and investigating reliability and associated factors of QBA with term lists, and additionally evaluating welfare in terms of the emotional state, were published for donkeys [7], cattle [8,9], pigs [10], and sheep [11], in particular. The advantages of QBA procedures with standardized term lists and calculations are the easier application and a straightforward interpretation of results by experienced assessors and experts, in particular when expressed within the welfare evaluation of the WQ-C12. The latter is given by the integrative handling of the collected QBA data, summing 20 terms in WQ-C12, allowing an easy calculation of a representative score at herd level and enabling an easier comparison between farms, systems, observers, and, furthermore, the identification of influencing factors for good and poor results. Consecutively, this might help to relate different management and farming practices to QBA results and add distinct information to a holistic welfare assessment [12] in dairy calves made by clinical examination and quantitative behavior observations as shown earlier for beef cattle [13]. Recently, such a QBA criterion score (WQ-C12) provided information transferrable to consumers, as well as valuable feedback to the farmer wishing to implement improvements [8,14]. 

In the past years, the welfare assessment in dairy cow systems focused only on lactating cows, neglecting the fact that also young calves and heifers are kept in large numbers on-farm and excluding them from welfare assessments, with a minor exception by Gratzer et al. [15]. Not only is the female offspring of particular high value for the farmer and the production systems’ economy, but at the same time the number of affected animals itself implies that they should be included in a welfare assessment, being representative of the farm and production system as a whole. Furthermore, in common dairy farming practice calves and cows are still forcedly separated after a few hours or days, both in organic and conventional systems. It could therefore be interesting to investigate the welfare state of the calves from a holistic point and emotional state, particularly seeing today’s need for alternatives to the early cow–calf separation [16]. In Norway, [17] the human–animal relationship in dairy calves was investigated using 31 QBA terms due to a missing standard procedure for dairy cows, fattening cattle, and veal calves [12] to describe dairy calves’ body language in handling situations by stockpeople. 

Calf husbandry practices in dairy farming follow some basic characteristics across Europe, such as the early (0–48 h) separation from the cow, the initial single or double penning followed by group housing at a later age, and very similar feeding strategies. However, at farm level the two included countries, Italy and Denmark, show great variation in structure and management regarding the outdoor or indoor housing of the calves, the access to pasture, lying comfort, health surveillance, the size of the farm both in number of animals and revenue, the kept breeds, or the available manpower. Some of these farm factors could potentially influence the emotional state of the calves and could be used in an analysis together with QBA results. Herd size is one of the interesting factors as this shows the greatest variation both within and between the two countries. The intensification of the dairy production over the past decades has left the Danish herds with an average herd size of 167 cows in 2015 compared to an average of 65.9 cows in 2000, rendering the larger herds with increased challenges regarding mortality [18] similar to their Nordic neighbors [19,20]. Additionally, herd size has also been associated with other management practices such as the choice of grazing or organic production status in smaller farms [21], an issue also evident in the Italian dairy production [22]. Larger production sites require a larger number of employees. In some farms, however, this only implies a greater number of animals per stockperson, which could negatively affect the welfare of the entire herd as discussed by Simon et al. [23]. Within the last decades, a vast number of non-natives being employed on Danish farms for varying time periods has led to a high turnover in manpower, challenging management in terms of language and cultural barriers and possibly different attitudes towards calf handling and rearing. To our knowledge, no previous studies have investigated the potential influence of these farm factors on the emotional state of calves. Likewise, studies evaluating observer agreement on QBA video material for the evaluation of a prespecified term list in dairy calves has not been published so far. Furthermore, information on emotional states on calves in dairy herds, especially in terms of aggregated scores (WQ-C12) at farm level, was desirable for future purposes.

Therefore, this study aimed at investigating a QBA procedure, including 20 terms, by extrapolating the main dimensions of the calves’ emotional states achieved by Principal Component Analysis (PCA) and testing reliability by means of comparing observer agreement on video material. Further, we performed the QBA aggregation procedure and integrated score calculation to achieve Welfare Quality criterion scores (WQ-C12) at farm level. Finally, we were investigating certain farm factors potentially related to the emotional state of dairy calves for identifying explanatory variables on WQ-C12.

## 2. Materials and Methods 

### 2.1. QBA Procedure

The WQ protocol for calves and heifers developed earlier [16] and the proposed QBA description was used for the on-farm assessments of emotional state. The proposed QBA was a term list with 20 descriptors, ’Active, Relaxed, Uncomfortable, Calm, Content, Tense, Enjoying, Indifferent, Frustrated, Friendly, Bored, Positively occupied, Inquisitive, Irritable, Nervous, Boisterous, Uneasy, Sociable, Happy, Distressed’, which was used to score behavior after an observation period of 20 min. Each term was scored on a 125 mm continuous scale (Visual Analogue Scale), where left represented the ‘minimum’ and right the ‘maximum’ point, by crossing each scale at a certain point fitting the observation. A very left position on the scale or a zero indicated that the expressed quality of the specific behavior/term was “entirely absent in any of the animals seen”, whereas the ‘Maximum’ at 125 mm stood for “the expressed quality of the specific behavior/term was constantly obvious across all animals seen during the observation” as was relevant in the case of herd observations [2]. Additionally, further considerations had to be integrated in the scoring by the observer, as the aim was also to imagine the respective endpoints of the scale as what was possible as an expression of a behavioral quality for the respective species for the given age and sex. Hence, the term “Inquisitive” would be at a different expected level in adult cows compared to calves. Training and testing of observers was performed to ensure calibration and the correct use of the scale for QBA. Initially, this included exchange of experiences amongst observers on different impressions from different husbandry systems or circumstances leading to minimum or maximum points on certain behavioral expressions, illustrating for their colleagues’ potential magnitudes and enhancing understanding of the potential dimensions. 

### 2.2. Video Sessions and Observers

Reliability testing of the chosen QBA term list was done by including eleven trained observers (7 females, 4 male) to a test panel which was asked to score QBA in 20 videos showing calves, in one go, with a length of 1–2 min each. All observers had a higher education at a university level in the field of Animal Science or Veterinary Medicine from across Europe, with both males and females being represented. All participants had a minimum experience of a week-long course on QBA in cattle (cows and calves), including on-farm and video practice, and score calculations with the WQ-QBA lists for dairy cattle and dairy calves. Prior to the video session, all the terms were discussed and calibrated again amongst all observers according to the definitions that can be found in Table 1. All observers scored the videos on QBA paper sheets with the above-mentioned term lists and transferred their scores (in mm) to a provided electronic data sheet.

### 2.3. QBA Application On-Farm, Observers, and Farms

Before the on-farm assessments started, four observers familiarized themselves with the terms of the above-mentioned list and their descriptions (Table 1), ensuring they understood the terms and used them in a similar context. Furthermore, the four observers, all female and veterinarians, applying the QBA were all previously trained for WQ in dairy cattle and welfare assessment of dairy calves including the new QBA system, respectively. They achieved sufficient agreement with a silver standard (previously trained and certified person) and each other (r > 0.7). For logistical reasons and limited resources of the study, each farm was visited by one observer only, observers assessed in their respective affiliated country. For the same reasons, farms were recruited by the observers themselves in their own countries, firstly dependent on farmers’ willingness to join voluntarily, and secondly observers tried to involve a high variation of farm sizes, breed, farming styles, and locations in order to potentially achieve a large variation of emotional states in calves. Therefore, the sample of farms ended up being a conveniently chosen one, an expected situation in on-farm studies, as nothing else was feasible. Nonetheless, it covered a wide range of different husbandry conditions for the calves, and therefore expectedly also a variety of potentially possible QBA results.

The on-farm assessments were carried out in dairy cow farms in Denmark (40) and in Italy (9), 31 conventional and 18 organic farms size varying from 106–608 lactating cows with an average of 48.2 calves per farm. Calves were mainly housed single or pairwise in the first month and later on moved to group housing. Breeds present on farms were either Holstein (25), other milk types such as Jersey or Brown Swiss (13), or mixed, meaning mixed breeds and/or mixed herds (11). In 13 of the farms, weaned calves grazed on pasture, farms keeping males had on average 12 bulls, and the number of full-time equivalent workers ranged from 1 to 3.5. The assessment was carried out in the early morning (around milking time for the cows, before morning feeding for the calves), as the first evaluation upon arrival at the farm. The calves at herd level were observed for 20 min in total, including all animals (male and female) aged 0–180 days present at the time of inspection. If animals were housed in several groups, the observation time between groups was split with a maximum of 8 observation points. In principle, animals were observed at group level even if housed individually or pairwise, in as large observational segments as possible and visible. After finishing the observations, the observers turned away from the animals, i.e., walked away from the animals to not observe any further, and made their scoring on a paper version of QBA.

### 2.4. Statistics

The data collected on-farm were analyzed using a Principal Component Analysis (PCA). The analysis was based on a correlation matrix, without rotation, and two components were extracted (after pre-exploration of an unlimited number of components and selection of eigenvalues >1 adjusted by the explained variances to avoid under/over factoring). Analyses were performed in R [24] using the libraries rcmdr, psych, Deducer, DeducerExtra. The dataset was sent to an external researcher, experienced with QBA and PCA, revealing the same PCA results and Graph of terms with a different statistical program (Minitab) to reassure correct methodology.

Accordingly, scores attributed to the calves by the 11 observers during the video’s administration were submitted to PCA analysis using a correlation matrix with no rotation. The PCA scores attributed to the 20 videos on the first two main Principal Components were tested for interobserver reliability using Kendall Correlation Coefficient W. Kendall W values can vary from 0 (no agreement at all) to 1 (complete agreement), with values higher than 0.6 showing substantial agreement. Subsequently, the interobserver reliability for each descriptor separately was calculated using the intraclass correlation coefficient (ICC).

### 2.5. Procedures with the On-Farm QBA Scores

The PC1-score, obtained from the PCA for each farm, was analyzed as a dependent variable in a linear regression model using the 20 terms as predictors to estimate the weights (reported as “estimates” in Table 1) according to the approach by Budaev [25]. The WQ criterion score (WQ-C12), defined in the WQ cattle protocol [12], was calculated using the following formula:

(1) $$WQ-C_{12}=constant+\;\overset{20}{\underset{k=1}{\sum }}w_{k}\;N_{k}$$

where *N_k_* is the value (in mm) obtained by a farm for a given term k, *w_k_* and the weight (= estimate, given in Table 1) attributed to a given term k ‘constant’, a fixed value for each farm (= intercept, given in Table 1)

The WQ-C12 was used for the interpretation of the calves’ welfare state. This index of the ‘Emotional state’ was analyzed applying a linear model and considering given farm characteristics and managerial choices as predictors (factors). The factors that were constant for every farm with no variation (e.g., age at separation from cow, if born in a calving box) or with too much missing information (>80%, e.g., bedding type of calves) were dropped out. The following factors could be considered for the analysis: housing outdoors or indoors of young calves (HOUSING_Y) and older calves (HOUSING_O) respectively, if weaned calves were on pasture or not (WEANED_PASTURE), the number of calves (CALVES_NO), cows (COWS_NO), young heifers (NO_YOUNGSTOCK) and bulls (NO-BULLS) on the farm, the prevalent cattle breed (BREED), the farming style as ‘organic’ or ‘conventional’ (FARMSTYLE), and how much manpower was available (MANPOWER). After pre-elimination of correlated factors, a back/forward procedure based on AIC revealed the best fitting model. Residuals were graphically checked using the function qqplot in R. 

## 3. Results

### 3.1. PCA for Video and On-Farm Data

Analysis of the video data looking into the agreement of 11 different observers, revealed good overall agreement (Cronbach’s Alpha 0.83) between observers on the QBA for dairy calves and an ICC of 0.83. Overall explained variance of the PCA was 29.15% for PC1 and 16.34% for PC2, with an eigenvalue of 5.83 and 3.39, respectively. Agreements between observers for each descriptor were good for 14 terms (Cronbach’s Alpha 0.74–0.97) and moderate for five terms (Cronbach’s Alpha 0.46–0.64), the term ‘distressed’ couldn’t’ be analyzed (further details in Table 2).

**Figure 1:**
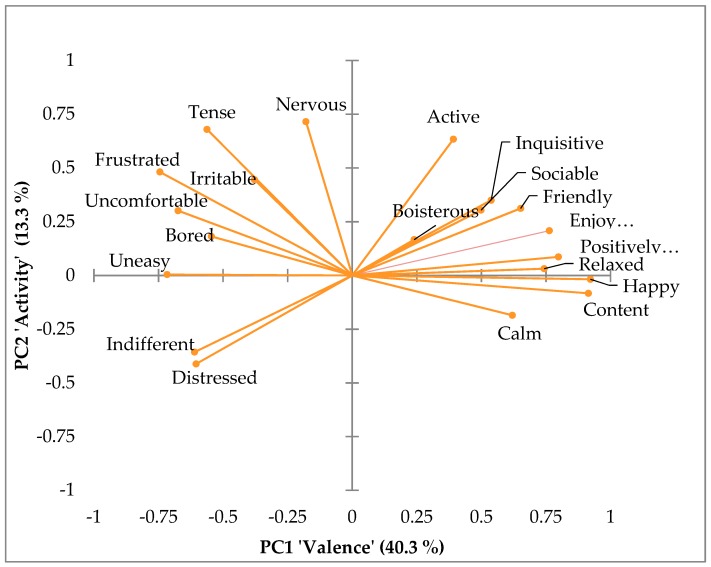
Two-dimensional loading plot of the Principal Component Analysis (PCA) results showing the distribution of all 20 terms describing the quality of calf behavior on PC1 (Valence) and PC2 (Arousal) as vectors.

PCA for the on-farm data summarized QBA descriptors on two main components, PC1 and PC2, with eigenvalues of 8.065 and 2.662, explaining 40.3% and 13.3% of the variation, respectively. Farm PC1 scores ranged between −2.67 and 1.49 and PC2 scores from –2.17 to a maximum of 2.42. The PC1 and PC2 loadings for the terms can be seen in detail in Table 1, and their distribution in the four quadrants in Figure 1. 

**Figure 2:**
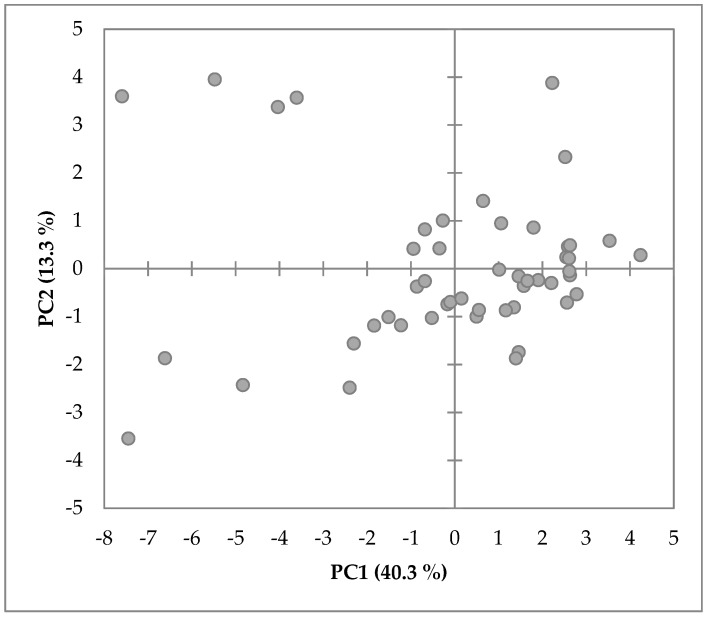
Two-dimensional loading plot of the PCA showing the distribution of the individual farm results of all 49 farms for PC1 and PC2.

Descriptors’ loadings with the highest positive values on PC1, summarized as ‘Valence’, were: ‘Relaxed’, ‘Calm’, ‘Content’, ‘Enjoying’, ‘Friendly’, ‘Positively occupied’, ‘Inquisitive’, ‘Sociable’, and ‘Happy’, loaded with a value greater or equal to 0.5. Furthermore, descriptors ‘Uncomfortable’, ‘Tense’, ‘Indifferent’, ‘Frustrated’, ‘Bored’, ‘Uneasy’, and ‘Distressed’, with a value smaller or equal to −0.56, represented the opposite direction. PC2, described as ‘Activity’, revealed ‘Active’, ‘Tense’, ‘Frustrated’, and ‘Nervous’ with values greater or equal to 0.48 as the most positive terms, and ‘Calm’, ‘Indifferent’, and ‘Distressed’ with values smaller or equal to −0.36 on the negative dimension (Table 1). On an individual level, farm results showed great dispersion across the two dimensions, as can be seen in the PCA graph ‘farm results’ in Figure 2, thus indicating that a discrimination of farms with two dimensions is satisfactory and sensitive enough to picture different emotional states of calf herds.

### 3.2. Welfare Quality Scores for the Emotional State of Calves and Associated Factors

The PC1 for each farm, translated to the WQ-criterion score (WQ-C12) gave a mean of 51.1 ± 9.0 points (0 points = worst to 100 points = excellent situation), indicating neutral states (on average) in the involved farms. However, as farm scores ranged from 28.84 to 66.19 points, the average was not representing the actual emotional state for all calf herds included in the study. Results of investigating between-farm variance by modeling WQ-C12 results with certain farm factors revealed that CALVES_NO, FARMSTYLE, and BREED stayed in the model, explaining WQ-C12 best (*p*-value < 0.05; r^2^_adjusted_: 0.18). Farms following the organic style achieved significantly higher estimates (+6.77; *p* < 0.01). In terms of farm size, every additional calf was revealing a significant, but small, positive effect (+0.05; *p* < 0.05). For ‘BREED’, effects were not significant and in our study. Holsteins had the highest (+3.75; *p* = 0.21) and Jersey the lowest estimates (−1.75; *p* = 0.61). 

## 4. Discussion

### 4.1. PCA and Achieved Dimensions

Firstly, this study aimed at investigating the dimensions created by Principal Component Analysis (PCA) using 20 terms describing different quality of behavior in dairy calves. Like other studies applying this procedure, PC1 was found to summarize terms describing ‘Valence’ and PC2 ‘Activity’, comparable to PC1 ‘Mood’ and PC2 ‘Activity’ for donkeys [7], cows [6], or pigs [10]. The distribution of certain terms across the four quadrants, and their meaningful loadings (>0.5) on PC1 and PC2 [25], also fitted the expressed behavior. Amongst others, the term ‘Frustrated’, loading very negatively on ‘Valence’ but positively on ‘Activity’, was well aligned with the expectations when picturing the behavioral modulation in young calves using this set of terms. Furthermore, the defined components can be seen as a good summary of the overall emotional state, as terms that are close in their expressed quality also go well together in groups across the quadrants, such as ‘Happy’ and ‘Content’, or ‘Indifferent’ and ‘Distressed’. There was only one term, ‘Boisterous’, not loading substantially on one of the two axes, indicating that it is not substantially needed to describe the emotional states of calves. Therefore, it could be argued to exchange it with another term or leave it out and redo the analysis. On the other hand, there might be farms in the future where this term will show a greater response, as it should be seen in the light of our case-to-variable ratio being 2.25, lower than reported from other studies by Budaev [25]. Besides that, breed differences in temperament might be causing this vaguer response. Coming from different countries and with different linguistic backgrounds, the team was communicating and scoring QBAs in English, giving room for slightly different interpretations of the term ‘Boisterous’, especially in young animals. This might be mitigated in the future by the intensive use of written QBA descriptors and definitions, such as those published for donkeys by Minero et al. [7] also seen in Table 1 in this paper. This approach would probably be helpful but cannot replace training and live agreement between observers. However, as shown by the weights that were assigned to ‘Boisterous’, it did not have a major impact on WQ-C12 and should be kept in the term list until further studies and more data are available. A similar argument could be done for the negative term ‘Distress’, which is a severe condition and thus was not recorded for the majority of observers in this study but would be very useful in case QBA is applied in extremely poor welfare conditions or discriminating ones.

### 4.2. Agreement of Observers in Video Study

For most of the terms, it seems that the current training regime and previous knowledge of QBA amongst the observers was sufficient for a good agreement. However, when looking at the terms with low agreement, which to a large extent described negative states such as ‘Indifferent’, ‘Frustrated’, ’Irritable’, or ’Uneasy’, it seems that observers had recognized different valences. Disagreement can have two reasons—different perceptions or different scorings. With regard to video sessions, well known limitations are the quality of the footage and the restricted viewing angle and overall impression towards the animal an observer has. In short clips, it might be difficult for some observers to focus immediately on the animals’ demeanor. However, short clips are preferred to avoid observers suffering from fatigue throughout a long video session, since a minimum number of videos is necessary for the analysis. 

A further source of disagreement is a very low or disguised level or absence of a certain term throughout videos, or unclear demarcation of terms. When confronting the observers with the inter-observer reliability of the video session, there were some remarks that some video footage was not good enough to see a lot of the body language of the animals. This might have caused the disagreement and highlights the importance of the footage quality. At the same time, it underlines the importance of training the second source of errors occurring when the scoring is not performed properly—what cannot be seen in the animals should also not be scored (or estimated instead). A general point in scoring QBA is the reference for the line from min to max that some observers build up with experience, to classify the present animals with respect to a certain term. In this video session this was maybe difficult for some observers, in particular if there was limited experience with a huge variety of emotional states in animals, and it might be that this aspect needed more attention in the training material. 

For proper scoring, observers also needed to be trained for the occasion that there might be different animals showing different graded valences. To what extent observers were able to implement this cannot be analyzed, and it therefore remains a potential source of the extent of disagreement in the above-mentioned terms. However, as the main amount of terms was scored with good agreement, it has been shown that agreement in principle can be trained and is possible. Future training sessions should therefore focus on terms with so far lower agreement and provide excellent video footage.

### 4.3. Emotional State of Dairy Calves on the Farms

As stated earlier, this was the first study for dairy calves aggregating the PC1 score to a WQ-C12, and therefore scaling QBA results from 0 (poor) to 100 (excellent) points on a welfare scale. Therefore, we lack direct comparison. In any case we can say the farms in this study reached an average neutral to slightly positive welfare state, based on the expert opinions of Welfare Quality [12]. Comparing PC1 scores of our study with veal calves from 24 Italian farms [4] showed a larger range for the PC1 (−5.08 to 3.88). Translated to WQ-C12, this would correspond to 13.6 to 86.9 points and an average of 52.5, pointing towards a neutral to positive emotional state. Another QBA in 63 beef bull farms with three assessments per farm, using almost the same terms as for the dairy calves, was revealed average PC1 scores ranging from −4.8 to 4.8. This corresponds to 15.2 to 93.2 points for WQ-C12, with an average of 48 points, stating a neutral emotional state in beef bulls [8]. Despite having a larger sample size in the present study than the compared veal studies, but lower sample size than the beef studies, a lower range in WQ-C12 was found. This might be explained by the fact that Danish dairy calf husbandry, which was finally dominant in this study, was more uniform and scorings were therefore more homogenous than in Italian veal calves and beef bulls from Austria, Germany, and Italy [4,8]. However, QBA was still able to discriminate between farms amongst the dairy calf herds of this study, furthermore corresponding to certain farm factors, which is arguing for a large enough variation and sensitivity of the defined terms list.

### 4.4. Associated Factors on the Welfare Quality Scores for Emotional State of Calves

Finally, as mentioned before, we were investigating certain explanatory farm factors and their potential association with the emotional state of dairy calves at farm level. Positive effects on the emotional state of dairy calves were found by herd size (i.e., number of calves), organic production style, and breed. However, breed had no significant effect in the univariable analyses. Nonetheless, breed can be seen in relation to herd size, as the larger Danish herds most commonly are Holstein herds, which was also reflected by the included study herds. Additionally, a large number of Jersey bull calves are culled shortly after birth, leaving only heifer calves on the dairy farms for assessment. The associations found in the present study are well aligned with other findings, due to the effects of organic production setup. Pairwise housing and group housing of calves are mandatory in organic farms, which lead to decreased fear levels [26,27]. Furthermore, enhancement in social interactions is aiding calves during the feed uptake in the transition period at weaning [28,29].

Studies investigating potential risk factors for an aggregated emotional state in cattle farms are rare to our knowledge. Brscic et al. [4] found differences in the descriptive terms ‘Active’ and ‘Lively’ according to the housing form of veal calves at the age of 3 weeks (single vs. group housed). In contrast to that, the present study could not find an influence of the type of housing in our study, neither in very young calves nor older ones. Ellingsen et al. [17] analyzed effects of four different handling styles of stockperson (‘Calm/Patient’, ‘Dominating/Aggressive’, ‘Positive interactions’, and ‘Insecure/Nervous’) and succeeded in proving an influence on positive and negative moods of dairy calves. Although stockpersons’ handling in our study was not observed due to study limitations, it would be valuable to include this in future studies on risk factors of emotional state and would probably lead to a higher explained variance in the model. The aspect of including stockmanship was also supported by the study from Norwegian colleagues [30], who investigated effects of farm factors and stockpersons’ handling style on QBA in goats. Findings included that the positive attitude towards petting goats significantly related negatively to the QBA term ‘Aggressive’ and positively to the term ‘Inquisitive/Interested’. In this study, we have also made farmer questionnaires with some of the farmers regarding their attitudes towards Animal Welfare, which is currently under investigation and will be part of our next study.

## 5. Conclusions

We conclude that with the 20 terms used, it is possible to achieve good overall observer agreement with trained persons. However, analysis pointed out that some terms would profit from improved video training of observers for a common understanding. We observed a relatively high portion of explained variation (54.0%) on PC1 (‘Valence’) and PC2 (‘Activity’) providing a differentiated view on the emotional state of calves. WQ-C_12_-scores were pointing at a neutral to positive emotional state, of which 18% of the variance could be related to number of calves on a farm, farming style, and prevalent breed, leaving enough potential for further investigations into which factors on farm might influence the emotional state.

## Figures and Tables

**Table 1:** QBA fixed-term list (20 terms) with definition of terms, loadings of Principal Component 1 and 2 (PC1 and PC2) and extracted weights for the simplified score aggregation.

Terms (Factors)	Definition of Term	PC1	PC2	Weights (Estimate)
(Intercept) = ‘constant’				−2.031
Active	Engage in an activity in a conscious manner, regardless of motor activity; can be resting, while attentive	0.39	0.63	0.001792
Relaxed	Body language is at ease, animals seem not stressed	0.74	0.03	0.003648
Uncomfortable	Animals display physical or mental discomfort in the given situation, may show attempts to avoid source of discomfort	−0.67	0.30	−0.006389
Calm	Even-tempered, still and quiet in the performance of activities	0.62	−0.19	0.003380
Content	Animals express overall contentment with their life situation and seem mentally balanced, in control of their constitution	0.91	−0.08	0.004339
Tense	Rigid postural or /and facial expression, muscle strains visible, stiff body posture or movements	−0.56	0.68	−0.009337
Enjoying	Express satisfaction in the given situation and occupation	0.76	0.21	0.003411
Indifferent	Animals are aware of their environment and stimuli, but do not engage in activities or react	−0.61	−0.36	−0.003649
Frustrated	Motivation cannot be satisfied, may lead to compulsive or replacement behavior	−0.74	0.48	−0.009898
Friendly	Animals display friendliness towards other animals and humans	0.65	0.31	0.003293
Bored	animals are idling or active without any purpose, no motivation detectable to engage in any kind of activity (Results of prolonged lack of stimuli)	−0.55	0.18	−0.003098
Positively occupied	Animals actions express that they “like to do what they do”in a given situation	0.80	0.09	0.003530
Inquisitive	Desire to perform active investigation of surroundings or conspecifics on the hunt for new experiences and stimuli	0.54	0.35	0.002364
Irritable	animals are easily upset and irritated or agitated	−0.38	0.45	−0.006465
Nervous	Elevated level of arousal and vigilance, might be combined with restlessness and body movements	−0.18	**0.72**	−0.002303
Boisterous	Heedless, reckless behavior without any sign of aggression	0.24	0.17	0.001505
Uneasy	Physically or mentally troubled, long-term state of discomfort	−0.72	0.00	−0.006855
Sociable	Actively seeking for social engagement	0.50	0.30	0.002185
Happy	Displaying excitement, joy and pleasure in a given situation	**0.92**	−0.02	0.004075
Distressed	Comprised adaptability resulting in incapability of action; animals resign, withdraw completely; close to death	−0.60	−0.41	−0.009291

Highest loading for each term is typed in bold.

**Table 2:** Cronbach’s Alpha, intraclass correlation coefficient (ICC), and number of agreements between observers for each descriptor used by the observers to assess the 20 videos.

Descriptor	Cronbach’s Alpha	ICC	No. Observers	Significance
active	0.89	**0.84**	11	<0.001
relaxed	0.74	0.59	11	<0.001
uncomfortable	0.77	**0.74**	11	<0.001
calm	0.84	**0.75**	11	<0.001
content	0.82	**0.75**	11	<0.001
tense	0.83	**0.78**	11	<0.001
enjoying	0.90	**0.88**	11	<0.001
indifferent	0.46	0.29	11	0.021
frustrated	0.49	0.44	11	0.011
friendly	0.88	**0.86**	11	<0.001
bored	0.87	**0.80**	11	<0.001
positively occupied	0.93	**0.90**	11	<0.001
inquisitive	0.64	0.54	10	<0.001
irritable	0.58	0.54	10	0.002
nervous	0.80	**0.76**	11	<0.001
boisterous	0.97	**0.97**	11	<0.001
uneasy	0.52	0.50	11	0.007
sociable	0.91	**0.90**	11	<0.001
happy	0.86	**0.75**	11	<0.001
distressed	-	-	11	- ^1^

^1^ couldn’t be analyzed as eight observers scored 0mm for the term. ICC values ≥0.60 are bold typed.
